# Incidence of postpartum infection, outcomes and associated risk factors at Mbarara regional referral hospital in Uganda

**DOI:** 10.1186/s12884-018-1891-1

**Published:** 2018-06-28

**Authors:** Joseph Ngonzi, Lisa M. Bebell, Yarine Fajardo, Adeline A. Boatin, Mark J. Siedner, Ingrid V. Bassett, Yves Jacquemyn, Jean-Pierre Van geertruyden, Jerome Kabakyenga, Blair J. Wylie, David R. Bangsberg, Laura E. Riley

**Affiliations:** 1 Department of Obstetrics and Gynecology, Mbarara University of Science and Technology, P.O. Box 1410, Mbarara, Uganda; 2 Global Health Institute, University of Antwerp, Antwerp, Belgium; 3 Division of Infectious Diseases, Massachusetts General Hospital, Boston, MA USA; 4 Massachusetts General Hospital Center for Global Health, Boston, MA USA; 5 Department of Obstetrics and Gynecology, Massachusetts General Hospital, Boston, MA USA; 6 Institute of Maternal Newborn and Child Health, Mbarara University of Science and Technology, Mbarara, Uganda; 7 Oregon Health & Science University-Portland State University School of Public Health, Portland, OR USA

**Keywords:** Incidence, Risk factors, Postpartum, Uganda, Resource limited, Pregnant women, Labor, Africa, Infection, Puerperal sepsis

## Abstract

**Background:**

There is a paucity of recent prospective data on the incidence of postpartum infections and associated risk factors in sub-Saharan Africa. Retrospective studies estimate that puerperal sepsis causes approximately 10% of maternal deaths in Africa.

**Methods:**

We enrolled 4231 women presenting to a Ugandan regional referral hospital for delivery or postpartum care into a prospective cohort and measured vital signs postpartum. Women developing fever (> 38.0 °C) or hypothermia (< 36.0 °C) underwent symptom questionnaire, structured physical exam, malaria testing, blood, and urine cultures. Demographic, treatment, and post-discharge outcomes data were collected from febrile/hypothermic women and a random sample of 1708 normothermic women. The primary outcome was in-hospital postpartum infection. Multivariable logistic regression was used to determine factors independently associated with postpartum fever/hypothermia and with confirmed infection.

**Results:**

Overall, 4176/4231 (99%) had ≥1 temperature measured and 205/4231 (5%) were febrile or hypothermic. An additional 1708 normothermic women were randomly selected for additional data collection, for a total sample size of 1913 participants, 1730 (90%) of whom had complete data. The mean age was 25 years, 214 (12%) were HIV-infected, 874 (51%) delivered by cesarean and 662 (38%) were primigravidae. Among febrile/hypothermic participants, 174/205 (85%) underwent full clinical and microbiological evaluation for infection, and an additional 24 (12%) had a partial evaluation. Overall, 84/4231 (2%) of participants met criteria for one or more in-hospital postpartum infections. Endometritis was the most common, identified in 76/193 (39%) of women evaluated clinically. Twenty-five of 175 (14%) participants with urinalysis and urine culture results met criteria for urinary tract infection. Bloodstream infection was diagnosed in 5/185 (3%) participants with blood culture results. Another 5/186 (3%) tested positive for malaria. Cesarean delivery was independently associated with incident, in-hospital postpartum infection (aOR 3.9, 95% CI 1.5–10.3, *P =* 0.006), while antenatal clinic attendance was associated with reduced odds (aOR 0.4, 95% CI 0.2–0.9, *P* = 0.02). There was no difference in in-hospital maternal deaths between the febrile/hypothermic (1, 0.5%) and normothermic groups (0, *P* = 0.11).

**Conclusions:**

Among rural Ugandan women, postpartum infection incidence was low overall, and cesarean delivery was independently associated with postpartum infection while antenatal clinic attendance was protective.

## Background

Postpartum infection is a leading cause of maternal mortality worldwide. Approximately five million cases of pregnancy-related infection occur every year globally, and approximately 75,000 result in death [1, 2]. Infection incidence is higher in low-resource settings, and many infection-related maternal deaths are preventable [1, 2]. Postpartum infections are a subset of maternal infections occurring between delivery and the 42nd day postpartum [3]. The most common postpartum infections include endometritis (puerperal sepsis), urinary tract infections, surgical site infections, blood stream infection and wound infections [3, 4]. In a retrospective study from Mbarara Uganda, puerperal sepsis accounted for 31% of maternal deaths, making it the most common cause of maternal mortality at that facility [5].

Most research on postpartum infections has occurred in high resource countries, where risk factors include poor intrapartum hygiene, low socioeconomic status, primiparity, prolonged rupture of membranes, prolonged labor, and having more than five vaginal exams intrapartum [6]. In these settings, cesarean delivery appears to be the single most important risk factor for postpartum infection [3, 6]. In low-resource settings, risk factors for postpartum infection are poorly defined and may differ from high-resource settings due to patient, environmental and healthcare system factors [1]. In addition, most published studies from low-resource settings do not include microbiological confirmation of infection or infectious outcomes [3].

We performed a prospective cohort study to determine the incidence of postpartum infection among women with postpartum fever or hypothermia presenting for delivery or postpartum care at Mbarara Hospital. In addition, the study compared clinical outcomes between the fever/hypothermic group and the normothermic group, and examined risk factors associated with incident fever/hypothermia and a composite postpartum infection outcome.

## Methods

### Study site and design

We conducted a prospective cohort study among women admitted for delivery or postpartum care at Mbarara Regional Referral Hospital (MRRH) in rural Uganda, in which a total of 4231 participants were enrolled. MRRH is both a regional referral and teaching hospital for Mbarara University of Science and Technology with 11,000 deliveries annually [7, 8]. Data from the entire cohort of participants was used to determine which risk factors were associated with developing a fever or hypothermia while hospitalized.

### Enrollment and data collection

Women admitted to the maternity ward at MRRH for delivery or within 6 weeks postpartum were screened for enrollment into the study. Participants providing written informed consent were followed by research nurses who measured vital signs including heart rate, blood pressure, respiratory rate and oral temperature approximately every 8 h starting immediately after delivery, as previously described [7]. Participants who had not been tested for human immunodeficiency virus (HIV) within the last 6 months were offered HIV testing. Women who did not understand English or Runyankole (the local language) or were incapacitated and their next-of-kin declined participation were excluded from the study. Questionnaires and laboratory results were entered into a Research Electronic Data Capture (REDCap) database [9].

### Sample size and planned analyses

The sample size was calculated at 3500 participants, the number of participants needed to detect a doubling of postpartum infection risk from 5 to 10%, comparing HIV-uninfected to HIV-infected women. An additional 721 participants were enrolled to examine secondary outcomes for a nested sub-study. All analyses described in this manuscript were planned a priori before cohort enrollment began.

### Sample collection and microbiology

A symptom questionnaire and structured physical exam developed by study investigators was administered to all participants febrile to > 38.0 °C or hypothermic < 36.0 °C by a trained research nurse. Febrile and hypothermic participants were tested for malaria using the SD Bioline Malaria Ag Pf/Pan rapid diagnostic test (RDT, Standard Diagnostics, Gyeonggi, Korea), provided a clean-catch urine sample, and had peripheral blood drawn aseptically into Becton Dickinson (BD) BACTEC (Becton, Dickinson and Company, Franklin Lakes, USA) aerobic, anaerobic and mycobacterial blood culture bottles. Blood culture bottles were processed at the Epicentre Mbarara Research Centre microbiology laboratory adjacent to MRRH as previously described [7].

### Additional data collection

All participants who were febrile and hypothermic and a randomly selected sample of normothermic participants underwent structured interview and chart review at time of hospital discharge. Random selection was performed using a random number generator function in Excel, with the goal of selecting five normothermic participants for every febrile/hypothermic participant. Participants were followed up by phone at two and 6 weeks postpartum using a structured questionnaire to determine maternal and infant vital status, interval healthcare encounters and antibiotic usage. Interview tools were created by study investigators and pilot-tested prior to study start. Data from all participants were included in the final analysis, even if participants had missing data on specific variables. Gestational age was defined by participant report or chart documentation of last normal menstrual period. Pregnancy losses at < 28 weeks’ gestation were defined as miscarriages.

### Defining postpartum infection

Postpartum endometritis (puerperal sepsis) was defined as infection of the genital tract with two or more of the following: pelvic pain, fever > 38.0 °C, abnormal vaginal discharge, and delay in the rate of reduction of the size of the uterus < 2 cm/day [10]. Upper, lower, and catheter-associated urinary tract infections (UTIs) were defined using standard published criteria as previously described [7]. Blood stream infection (bacteremia) was defined as growth of a potential pathogen in one or more blood culture bottles. The composite outcome of postpartum infection was defined as having one or more of the following: postpartum endometritis, UTI or bloodstream infection.

### Data analysis

This present study is the primary analysis of data collected in this prospective cohort study. Summary statistics were used to characterize the cohort. Demographic characteristics and outcomes were compared between febrile/hypothermic participants and normothermic participants using Chi-squared analysis for categorical variables and student’s t-test or Wilcoxon Ranksum for continuous variables. *P*-values < 0.05 were considered statistically significant. Separate multivariable logistic regression models were used to identify factors associated with development of fever/hypothermia, postpartum endometritis and the composite postpartum infection outcome. Predictor variables selected for potential inclusion in each model included published risk factors for postpartum fever and infection such as age, parity, employment, district of residence, comorbidities, number of vaginal exams in labor, and reported duration of labor. Additional variables were included if on bivariate analysis they demonstrated a correlation with the outcome of interest with a *P*-value < 0.1. Backwards stepwise elimination was used to create the final model, and all variables with *P*-values< 0.05 in the final model were considered significant independent predictors of the outcome. Data from all participants enrolled into the study were included in the final analysis regardless of whether the subjects were retained or later withdrew. If patients withdrew their consent, no further sample or data collection was performed. All analyses were performed using Stata software (Version 12.0, StataCorp, College Station, TX).

## Results

### Enrollment and demographics

**Fig. 1:**
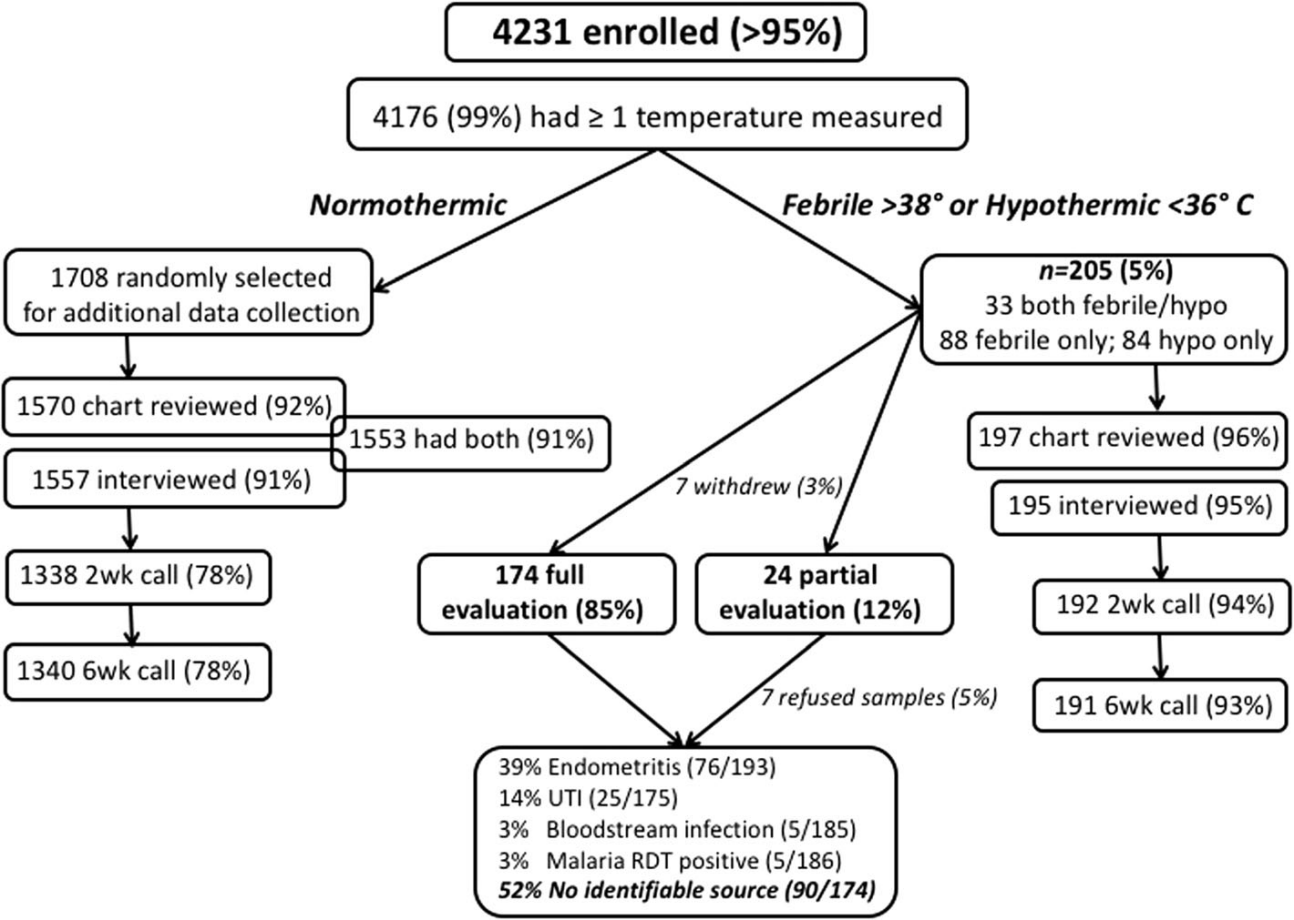
Enrollment, retention, clinical and laboratory testing for women enrolled into a prospective cohort study of postpartum infection at Mbarara Regional Referral Hospital, Uganda

Of all eligible women presenting to MRRH for care during the study period, over 99% (4235) were enrolled, four withdrew before data collection was performed, for a total enrollment of 4231. At least one temperature measurement was recorded for 4176 (99%) of participants, and two or more measurements were recorded for 2917 (69%, Fig. 1). Among the 4176 participants with a temperature recording, fever/hypothermia was recorded at least once in 205 (5%). Of these, 10 (5%) were missing interview or chart review and 14 (7%) were missing 2-week and 6-week follow up data. Of 1708 normothermic participants randomly selected for additional data collection, 138 (8%) and 151 (9%) were missing chart review and interview data, respectively. Two- and six-week follow up data was missing in 368 (22%) and 370 (22%) respectively (Fig. 1). Of those who underwent chart review and interview, missing data ranged from 0 to 8% for individual variables.

Among the 1913 febrile/hypothermic and randomly selected normothermic women, 1752 underwent interview and 1767 underwent chart review. The mean age was 25.2 years (standard deviation (SD) 5.5 years), 214 (12%) were HIV-infected, 874 (51%) delivered by cesarean, and 662 (38%) were primiparous (Table 1). The mean age of febrile/hypothermic participants was significantly younger than normothermic participants (23.5 versus 25.4 years, SD 5.5 years, *P <* 0.001)*.* Febrile/hypothermic participants were also less likely to reside within Mbarara (32% versus 45%, *P* = 0.001) or to be formally employed (41% versus 26%, *P* < 0.001). Febrile/hypothermic participants were more likely to be referred from another health facility (24% versus 12%, *P* < 0.001) and more likely to be primiparous (55% versus 36%, *P* < 0.001) than normothermic participants. Cesarean delivery was also significantly more common among febrile/hypothermic participants (81% versus 47%, *P* < 0.001). The proportion of HIV-infected did not differ between the two groups (12% each *P* = 0.86).

**Table 1:** Demographics, obstetric history and post-delivery care of study participants, comparing febrile and hypothermic participants to normothermic participants

Characteristic (*n* = 1913)	Normothermic number (%) (*n* = 1708)	Febrile or hypothermic number (%) (*n* = 205)	*P*-value*
*Sociodemographics*			
Age category			< 0.001
≤19	187 (12)	47 (24)	
20–34	1217 (79)	142 (73)	
> 34	135 (9)	6 (3)	
Residence in Mbarara municipality	664 (45)	58 (32)	0.001
Married	1452 (93)	177 (91)	0.19
No formal employment	925 (59)	145 (74)	< 0.001
Median household monthly income in UGx (USD)	150,000 (42)	100,000 (28)	0.10
HIV-infected	190 (12)	24 (12)	0.86
Referred from another health facility	183 (12)	48 (24)	< 0.001
*Pregnancy & Obstetric History*			
Admitted in labor	1503 (96)	183 (93)	0.046
Attended ≥4ANC visits	1101 (71)	138 (71)	0.99
Malaria prophylaxis during pregnancy (or taking TMP/SMX)	1434 (92)	175 (90)	0.26
Medical condition reported during pregnancy			
Syphilis or sexually transmitted infection	47 (3)	9 (5)	0.23
Urinary tract infection	47 (3)	3 (2)	0.24
Malaria	152 (10)	18 (9)	0.81
Diabetes, cardiac or renal condition	4 (0.3)	0 (0)	0.48
Total number of pregnancies			< 0.001
1	555 (36)	107 (55)	
2–4	799 (51)	71 (36)	
≥5	203 (13)	17 (9)	
Gestational age at delivery			0.45
Preterm (< 37 weeks)	140 (10)	22 (13)	
Term (37–42 weeks)	1199 (82)	131 (78)	
Post-term (> 42 weeks)	121 (8)	16 (9)	
Number of vaginal exams (self-reported)			0.32
0–4	1269 (89)	165 (91)	
≥5	162 (11)	16 (9)	
Delivery mode			< 0.001
Vaginal (spontaneous, breech or assisted)	814 (53)	37 (19)	
Cesarean	720 (47)	154 (81)	
Estimated duration of labor (mean hours, SD)	16.2 (SD 15)	19.7 (SD 17)	0.01
Singleton pregnancy	1505 (97)	188 (96)	0.37
*Post-Delivery Care*			
Peri-Cesarean antibiotic prophylaxis received	666 (99)	135 (99)	0.85
Urinary catheter placed	755 (48)	159 (82)	< 0.001
Catheter days (mean days, SD *n* = 914)	1.9 (1.0)	2.2 (1.3)	< 0.001
Duration of hospitalization (*n* = 1769; days, mean SD)	2.3 (2.1)	4.7 (5.9)	< 0.001

*HIV* human immunodeficiency virus, *UGx* Uganda Shillings, *USD* United States Dollar, *TMP/SMX* trimethoprim/sulfamethoxazole, *ANC* antenatal clinic, *SD* standard deviation

*Tests of association between cohort characteristics and temperature classification were performed using Chi squared, Fisher’s exact, and Student’s t-tests, where appropriate

### Evaluation of fever and hypothermia

Of 205 febrile or hypothermic participants, 174 (85%) completed physical exam and symptom questionnaire, provided blood and urine cultures, and underwent malaria RDT. Another 7/205 (3%) withdrew from the study, and 24/205 (12%) underwent partial evaluation: 7/205 (3%) refused sample collection and clinical evaluation but did not withdraw, 11/205 (5%) were missing urine culture, 1/205 (0.5%) was missing blood cultures, 3/205 (1%) refused sample collection but underwent clinical examination and 2/205 (1%) refused clinical examination and sample collection but completed the symptom questionnaire (Fig. 1).

### In-hospital postpartum infection incidence

Overall, 84/4231 (2%) of participants met criteria for one or more in-hospital postpartum infections. Endometritis was the most common, identified in 76/193 (39%) of women evaluated clinically. Of these 74 women with clinical endometritis and a recorded delivery mode, 61 (82%) delivered by cesarean, for a postpartum endometritis incidence of 7% among all recorded 875 cesarean deliveries. Twenty-five of 175 (14%) participants with urinalysis and urine culture results met criteria for UTI. Bloodstream infection was diagnosed in 5/185 (3%) participants with blood culture results. Another 5/186 (3%) were malaria RDT-positive. A diagnosis of cesarean surgical site wound infection was recorded in the chart for 5/205 (2%) febrile or hypothermic participants. The remaining 90/174 (52%) participants fully evaluated did not have a documented source of fever after our evaluation. The population attributable fraction (PAF) of postpartum infections due to cesarean delivery was 44%.

### In-hospital complications

Development of any in-hospital complication (including surgical site infection, re-admission to hospital, re-operation) was more common in febrile/hypothermic participants than normothermic participants (7% versus 1%, *P* < 0.001, Table 2). There was one participant who died in the hospital from the febrile/hypothermic group versus none in the normothermic group (*P* = 0.11). More febrile/hypothermic women than normothermic women had died by 6 weeks postpartum (3 versus 0 deaths, *P* = 0.002). Birth and perinatal outcomes were also worse in the febrile/hypothermic group including a significantly higher number of stillbirths (9% versus 3%, *P* < 0.001), and lower mean 5-min Apgar score (9.0 versus 9.6, *P* < 0.001), and neonatal or infant death within 6 weeks of life (12% versus 5%, *P* < 0.001, Table 2).

**Table 2:** Comparison of maternal and fetal outcomes between febrile/hypothermic participants and normothermic participants

Characteristic (*n* = 1913)	Normothermic number (%) (*n* = 1708)	Febrile or hypothermic number (%) (*n* = 205)	*P*-value*
*MATERNAL COMPLICATIONS* (*n* = 1726)			
Any of the following complications	13 (1)	13 (7)	< 0.001
Hysterectomy	1 (0.1)	0 (0)	1.0
Ruptured uterus	4 (0.3)	2 (1)	0.14
Blood transfusion	6 (0.4)	0 (0)	1.0
Re-operation	0 (0)	4 (2)	< 0.001
Death	0 (0)	1 (1)	0.11
Cesarean wound surgical site infection	3 (0.2)	7 (4)	< 0.001
Intensive care unit admission	0 (0)	1 (1)	0.11
Readmission to MRRH during postpartum period	12 (1)	17 (8)	< 0.001
Maternal death within 6 weeks	0 (0)	3 (2)	0.002
*MATERNAL CHART DIAGNOSES* (*n* = 1767)			
Premature rupture of membranes	19 (1)	8 (4)	0.002
Pre-eclampsia or eclampsia	14 (1)	4 (2)	0.13
Puerperal sepsis	2 (0.1)	14 (7)	< 0.001
Obstructed or prolonged labor	107 (7)	31 (16)	< 0.001
Antepartum hemorrhage	11 (1)	0 (0)	0.24
Chorioamnionitis	2 (0.1)	0 (0)	0.62
Malaria	2 (0.1)	1 (1)	0.22
*FETAL AND BIRTH OUTCOMES* (*n* = 1680)			
Outcome of singleton neonate by discharge			< 0.001
Miscarriage	2 (0.1)	0 (0)	
Stillborn	40 (3)	16 (9)	
Live birth	1431 (97)	168 (91)	
Live birth, in hospital death	9 (1)	1 (1)	
Singleton alive at discharge, dead by 2 weeks (*n* = 1415)	11 (1)	4 (2)	0.07
Live birth, multiple gestation (*n* = 106)	85 (96)	15 (88)	0.25
1-min Apgar score (*n* = 1648, mean, SD)	8.4 (1.8)	7.8 (2.6)	< 0.001
5-min Apgar score (*n* = 1642, mean, SD)	9.6 (1.8)	9.0 (2.8)	< 0.001
Singleton birth weight (kilograms)			0.01
< 2.5	83 (6)	14 (7)	
2.5–3.5	1048 (70)	145 (78)	
3.6–4.0	286 (19)	27 (14)	
> 4	73 (5)	1 (1)	
Twin/triplet birth weight < 2.5 kg (*n* = 106)	36 (41)	12 (71)	0.02
Stillbirth or neonatal death within 6 weeks (*n* = 1597)	65 (5)	22 (12)	< 0.001

*MRRH* Mbarara Regional Referral Hospital, *SD* standard deviation, *IQR* Interquartile range

*Tests of association between complications/outcomes and temperature classification were performed using Chi squared, Fisher’s exact, and Student’s t-tests, where appropriate

### Multivariable analysis of factors associated with postpartum outcomes

In multivariable logistic regression analysis, factors independently associated with postpartum fever/hypothermia included history of sexually transmitted infection (STI) during pregnancy (adjusted odds ratio [aOR] 4.0, 95% confidence interval [CI], 1.7–9.6) and cesarean delivery (aOR 2.9, 95% CI, 1.8–4.8, Table 3). Formal employment (aOR 0.5, 95% CI, 0.3–0.8) and multiparity (aOR 0.5, 95% CI, 0.3–0.7) were associated with reduced odds of postpartum fever/hypothermia. Factors independently associated with the composite in-hospital postpartum infection outcome (including confirmed diagnosis of UTI, endometritis or bloodstream infection) were cesarean delivery (aOR 3.9, 95% CI, 1.5–10.3) and increasing number of days admitted to hospital (aOR 1.2, 95% CI, 1.1–1.3). Attending antenatal care clinic ≥4 times was associated with reduced odds of postpartum infection (aOR 0.4, 95% CI, 0.2–0.9, Table 4).

**Table 3:** Univariable and multivariable logistic regression analysis of factors associated with postpartum fever/hypothermia among all study participants

	Univariable		Multivariable	
Characteristic	OR (95% CI)	*P*-value*	OR (95% CI)	*P*-value*
History of STI during pregnancy	1.5 (0.7–3.3)	0.29	4.0 (1.7–9.6)	0.002
Cesarean delivery	4.8 (3.3–7.0)	< 0.001	2.9 (1.8–4.8)	< 0.001
Number of days admitted to hospital	1.4 (1.3–1.5)	< 0.001	1.2 (1.1–1.4)	< 0.001
Formal employment	0.5 (0.3–0.7)	< 0.001	0.5 (0.3–0.8)	0.002
Multiparous	0.6 (0.4–0.7)	< 0.001	0.5 (0.3–0.7)	0.001
Residence in Mbarara municipality	0.6 (0.4–0.8)	0.001	0.7 (0.5–1.1)	0.11
Number of vaginal exams in labor	1.0 (0.9–1.1)	1.0	0.9 (0.8–1.0)	0.06
HIV-infected	1.0 (0.6–1.6)	1.0	1.0 (0.5–1.9)	0.94
Age	0.9 (0.9–1.0)	< 0.001	1.0 (0.9–1.0)	0.40
Number of hours in labor	1.0 (1.0–1.0)	0.08	1.0 (1.0–1.0)	0.95
Referred from an outside facility	2.6 (1.8–3.7)	< 0.001	1.5 (0.9–2.4)	0.10

*CI* confidence interval, *OR* odds ratio, *STI* sexually transmitted infection, *HIV* human immunodeficiency virus

*Tests of association between cohort characteristics and the presence or absence of postpartum fever or hypothermia were performed using univariable or multivariable logistic regression analysis

**Table 4:** Univariable and multivariable logistic regression analysis of factors associated with composite postpartum infection outcome among all cohort participants, including in-hospital confirmed diagnosis of urinary tract infection, endometritis or bloodstream infection

	Univariable		Multivariable	
Characteristic	OR (95% CI)	*P*-value*	OR (95% CI)	*P*-value*
Cesarean delivery	7.7 (3.9–15.1)	< 0.001	3.9 (1.5–10.3)	0.006
Number of days admitted to hospital	1.3 (1.2–1.4)	< 0.001	1.2 (1.1–1.3)	0.001
Attended antenatal care ≥4 times	0.7 (0.4–1.2)	0.20	0.4 (0.2–0.9)	0.02
Multiparous	0.3 (0.2–0.5)	< 0.001	0.5 (0.3–1.0)	0.06
Formal employment	0.6 (0.4–1.0)	0.04	0.7 (0.4–1.2)	0.20
Number of vaginal exams in labor	1.0 (0.9–1.1)	0.97	0.9 (0.8–1.1)	0.24
HIV-infected	1.0 (0.5–2.1)	0.91	1.4 (0.6–3.3)	0.49
Age	0.9 (0.8–0.9)	< 0.001	0.9 (0.9–1.0)	0.08
Number of hours in labor	1.0 (1.0–1.0)	0.26	1.0 (1.0–1.0)	0.66
Referred from an outside facility	2.3 (1.4–4.0)	0.002	1.1 (0.5–2.4)	0.75
Diagnosis of obstructed labor	2.5 (1.3–4.6)	0.005	1.4 (0.6–3.2)	0.40
Admitted to the floor at any time	0.4 (0.3–0.7)	< 0.001	0.8 (0.4–1.5)	0.45
Residence in Mbarara municipality	0.6 (0.4–1.0)	0.08	0.8 (0.4–1.5)	0.52
Number of days with a urinary catheter in place	1.8 (1.5–2.1)	< 0.001	1.1 (0.8–1.4)	0.53

*CI* confidence interval, *OR* odds ratio, *STI* sexually transmitted infection, *HIV* human immunodeficiency virus

*Tests of association between cohort characteristics and the presence or absence of postpartum composite outcome were performed using univariable or multivariable logistic regression analysis

Factors independently associated with development of in-hospital clinically-confirmed diagnosis of postpartum endometritis, which was the most common outcome amongst the infections, were cesarean delivery (aOR 2.7, 95% CI, 1.2–6.2) and increasing number of days admitted to hospital (aOR 1.2, 95% CI, 1.1–1.3). Multiparity was associated with reduced odds of postpartum endometritis (aOR 0.5, 95% CI, 0.2–1.0, Appendix 1).

## Discussion

In this prospective cohort of women presenting to a Ugandan regional referral hospital, among those who developed postpartum fever/hypothermia, cesarean delivery was the strongest independent risk factor for developing endometritis or a composite postpartum infection outcome. Other risk factors independently associated with postpartum infection included longer hospital stays and attending antenatal clinic fewer than the four visits recommended by 2015 Ugandan national guidelines. Therefore, efforts should be made to reduce the high proportion of cesarean deliveries, increase antenatal care attendance, reduce the number of days of admission and reduce the number of days of indwelling urethral catheters.

The incidence of postpartum fever or hypothermia in our cohort was 5%, and a source of infection was confirmed in 48% of those with documented fever or hypothermia, for a 2% overall incidence of confirmed in-hospital postpartum infection. The overall fever and infection incidence we report here is low. However, the most common infection among our participants was postpartum endometritis, and among cesarean deliveries we report an incidence of (7%), over 3-fold greater than estimates from high-resource settings (1.8–2.0%) [11–13]. Though infection incidence in Mbarara appears higher than European and North American estimates, comparing our findings to other low-resource settings is difficult. The reported incidence of postpartum endometritis in sub-Saharan Africa varies widely, likely due to differences in infection definition, surveillance, diagnosis, patient population and healthcare practices. One study at Uganda’s largest referral hospital, where HIV prevalence is 21%, reported 73/478 (15%) patients undergoing emergency cesarean delivery developed postpartum endometritis [14], more than double the 7% incidence reported here. The lower incidence of postpartum endometritis we report may reflect differences in practice, antibiotic use, and infection control procedures within Uganda. Also, the other Ugandan study was published in 2011, at a time when fewer HIV-infected women were on antiretroviral therapy, which could have led to higher infection rates. Historically, HIV has been associated with increased risks of postpartum sepsis, including postpartum endometritis [15]. Other studies from sub-Saharan Africa report postpartum endometritis in 1–17% after cesarean delivery [16–20], and our report of 7% incidence falls within this wide range. Though the incidence we report here is relatively low, postpartum infection may become more common in sub-Saharan Africa as a result of increasing cesarean delivery rates coupled with rising incidence of nosocomial infections [7].

We found UTIs in 14% and bloodstream infections in 3% of febrile/hypothermic participants. Comparisons of the incidence of UTI and bloodstream infections to other studies are difficult, as these infections are defined and reported inconsistently in the few other studies from sub-Saharan Africa [13, 14]. However, postpartum UTI incidence in some European studies is as low as 3% after cesarean delivery and 2% after vaginal delivery [21]. The difference in UTI incidence between our study and the other studies may be attributable to the fact that laboratory diagnosis of UTIs in our study was performed only for febrile and hypothermic participants, a group with a high likelihood of infection. It is also possible that cesarean delivery preparation and urinary catheter days may differ in other settings.

Report of sexually transmitted infection diagnosis during pregnancy, cesarean delivery, increasing number of hospital days, lack of formal employment and primiparity were independently associated with fever/hypothermia in our cohort. Predictors of postpartum fever in low-resource settings are not very well described in the literature, except that prolonged second stage of labor is a risk factor for postpartum fever [2]. Our report of postpartum fever associations with STIs and primiparity is not reported elsewhere in the literature, and merits further investigation.

Of note, birth and perinatal outcomes were overall worse in the fever/hypothermic group compared to the normothermic group. It is possible these differences reflect a pathological process present before delivery which could have contributed to poor fetal and neonatal outcomes. This is an area of investigation that should be explored to better understand maternal inflammatory and infectious contributors to stillbirth and early neonatal death and how to prevent postpartum infection.

Cesarean delivery was associated with the composite in-hospital postpartum infection outcome (including confirmed diagnosis of UTI, endometritis or bloodstream infection). In fact, in multivariable logistic regression models for each of the three outcomes (fever/hypothermia, endometritis, postpartum infection composite outcome), cesarean delivery was independently associated with each outcome with adjusted odds ratios of 2.7–3.9. This finding is consistent with other reports that postpartum infection is three times more likely to occur after cesarean section than after vaginal delivery [22]. In addition, the population attributable fraction of postpartum fever and postpartum infections due to cesarean delivery in our study was 44%. Our findings support previous research indicating that cesarean delivery is the most important risk factor for developing postpartum infection [3, 6, 23]. Our results should reinforce efforts to reduce cesarean delivery rates to appropriate levels to avoid preventable, cesarean-associated infections. In addition, antibiotic prophylaxis, hygienic delivery, and postpartum care conditions should continue to be emphasized as important factors mitigating infection risk. We also found that attending the recommended number of antenatal care visits (≥4 times during pregnancy at the time this study was conducted) was associated with reduced odds postpartum infection. Antenatal clinic interventions may help prevent postpartum infection through earlier detection and treatment of disease conditions, including sexually transmitted infections and UTI. Lastly, long hospital stays are a known risk factor for developing postpartum UTIs [23], and likely contribute to incident postpartum endometritis. Though prolonged hospitalization can result from fever or infection, it can also contribute to development of infection through increasing nosocomial transmission risk, prolonged exposure to invasive catheters and devices, and unhygienic conditions.

Strengths of our study include the prospective study design, large sample size, near-complete enrollment of eligible women seeking care at MRRH during the study period, and an in-depth clinical and microbiological evaluation of participants with suspected infection. Of note, at MRRH cesarean deliveries are performed under spinal anesthesia, and no cesarean or vaginal delivery participants had epidural anesthesia for labor analgesia. Epidural placement is one of the commonest causes of fever in labor and immediate postpartum period [24, 25], but does not confound the findings in our study.

Limitations of our study include reliance on chart diagnosis of surgical site wound infection, which was inconsistently documented. Though the initial study design did not include wound infection as part of the composite postpartum infection outcome, cesarean surgical site infection is a known cause of postpartum fever [26] and may account for a high proportion of fevers and hypothermia in participants with no other confirmed infectious source after our evaluation. We abstracted chart diagnosis of cesarean section surgical site infections but did not perform clinical or microbiologic evaluation of these infections. Lack of confirmation of cesarean wound infections is a limitation of our study since these are likely under-reported inpatient charts. In addition, due to resource constraints, we were unable to perform clinical or microbiological testing of normothermic participants and thus unable to determine the incidence of infection in the normothermic group. However, we expect that clinically significant in-hospital postpartum infections would include fever or hypothermia and therefore we believe we were unlikely to have missed significant infections in normothermic women. Prolonged rupture of membranes is a known risk factor for postpartum infection but was not directly measured in this study. We collected participant-reported duration of labor as one measurement of prolonged labor but we did not measure duration of membrane rupture directly. Lastly, at this regional referral hospital cesarean deliveries are common, accounting for 50% of all deliveries in this study. Though the cesarean delivery rate is high at MRRH, 50% may overestimate the true cesarean delivery rate due to early discharge and non-enrollment of some women delivering vaginally. We documented whether a woman was prescribed antibiotics on the same day as her cesarean section procedure, but we were unable to confirm whether these were given, nor determine the timing of the prescription relative to the procedure. Future research should address infections occurring after hospital discharge, incident in-hospital and post-discharge surgical site infection, and the impact of prophylactic antibiotics on incident infection and development of antimicrobial resistance.

## Conclusions

In our low-resource setting, cesarean delivery is independently associated with risk of incident in-hospital postpartum fever/hypothermia, endometritis and a composite postpartum infection outcome. Other independent risk factors for postpartum infection include longer hospital stays and attending antenatal clinic fewer than the recommended four visits. Efforts at reducing the high proportion cesarean deliveries, increasing antenatal care attendance, and reducing the number of days admitted and days of urethral indwelling catheter need to be optimized. With the high proportion of women with no identifiable source of infection, there is need for better bedside diagnostic means and antimicrobial evidence-based prescription.

## Appendix

**Table 5:** Univariable and multivariable logistic regression analysis of factors associated with in-hospital clinically-confirmed diagnosis of postpartum endometritis in postpartum infection study participants

	Univariable		Multivariable	
Characteristic	OR (95% CI)	*P*-value*	OR (95% CI)	*P*-value*
Cesarean delivery	5.4 (2.9–10.1)	< 0.001	2.7 (1.2–6.2)	0.02
Number of days admitted to hospital	1.3 (1.2–1.4)	< 0.001	1.2 (1.1–1.3)	< 0.001
Multiparous	0.3 (0.2–0.6)	< 0.001	0.5 (0.2–1.0)	0.05
Formally employed	0.6 (0.4–1.0)	0.06	0.6 (0.3–1.1)	0.11
Number of vaginal exams in labor	1.0 (0.8–1.1)	0.76	0.9 (0.7–1.1)	0.20
HIV-infected	1.4 (0.7–2.6)	0.37	1.8 (0.0–4.1)	0.18
Age	0.9 (0.9–1.0)	< 0.001	0.9 (0.9–1.0)	0.16
Number of hours in labor	1.0 (1.0–1.0)	0.23	1.0 (1.0–1.0)	0.09
Referred from an outside facility	2.5 (1.4–4.4)	0.001	1.3 (0.6–2.7)	0.55
Diagnosis of obstructed labor	2.3 (1.2–4.5)	0.02	1.3 (0.5–3.1)	0.56
Admission to the floor at any time	0.5 (0.3–0.9)	0.009	0.8 (0.4–1.6)	0.57

*CI* confidence interval, *OR* odds ratio, *STI* sexually transmitted infection, *HIV* human immunodeficiency virus

*Tests of association between cohort characteristics and the presence or absence of postpartum endometritis were performed using univariable or multivariable logistic regression analysis

## Abbreviations

ANC: Antenatal clinic
CAUTI: Catheter-associated urinary tract infection
CI: Confidence interval
HIV: Human immunodeficiency virus
MRRH: Mbarara Regional Referral Hospital
OR: Odds ratio
RDT: Rapid diagnostic test
SD: Standard deviation
STI: Sexually transmitted infection
TMP/SMX: Trimethoprim/sulfamethoxazole
UGx: Ugandan shillings
UTI: Urinary tract infection
